# Structural and biochemical insights into Zn^2+^-bound EF-hand proteins, EFhd1 and EFhd2

**DOI:** 10.1107/S2052252523001501

**Published:** 2023-03-01

**Authors:** Sang A Mun, Jongseo Park, Jung Youn Kang, Taein Park, Minwoo Jin, Jihyeong Yang, Soo Hyun Eom

**Affiliations:** a School of Life Sciences, Gwangju Institute of Science and Technology, Gwangju, Republic of Korea; b Steitz Center for Structural Biology, Gwangju Institute of Science and Technology, Gwangju, Republic of Korea; c Department of Chemistry, Gwangju Institute of Science and Technology, Gwangju, Republic of Korea; University of Michigan, USA

**Keywords:** EFhd1, EFhd2, crystal structure, EF-hands, actin-binding/bundling protein

## Abstract

EF-hand proteins, which contain a Ca^2+^-binding EF-hand motif, are involved in regulating diverse cellular functions. Ca^2+^ binding induces conformational changes that modulate the activities of EF-hand proteins. Moreover, these proteins occasionally modify their activities by coordinating metals other than Ca^2+^, including Mg^2+^, Pb^2+^ and Zn^2+^, within their EF-hands. EFhd1 and EFhd2 are homologous EF-hand proteins with similar structures. Although separately localized within cells, both are actin-binding proteins that modulate F-actin rearrangement through Ca^2+^-independent actin-binding and Ca^2+^-dependent actin-bundling activity. Although Ca^2+^ is known to affect the activities of EFhd1 and EFhd2, it is not known whether their actin-related activities are affected by other metals. Here, the crystal structures of the EFhd1 and EFhd2 core domains coordinating Zn^2+^ ions within their EF-hands are reported. The presence of Zn^2+^ within EFhd1 and EFhd2 was confirmed by analyzing anomalous signals and the difference between anomalous signals using data collected at the peak positions as well as low-energy remote positions at the Zn *K*-edge. EFhd1 and EFhd2 were also found to exhibit Zn^2+^-independent actin-binding and Zn^2+^-dependent actin-bundling activity. This suggests the actin-related activities of EFhd1 and EFhd2 could be regulated by Zn^2+^ as well as Ca^2+^.

## Abstract 2

The EF-hands within the crystal structures of the Zn^2+^-bound EFhd1 and EFhd2 core domains coordinate Zn^2+^ ions. EFhd1 and EFhd2 exhibited Ca^2+^- and Zn^2+^-independent actin binding and Ca^2+^- and Zn^2+^-dependent actin-bundling activity, and these suggest the possibility that both Ca^2+^ and Zn^2+^ act to regulate EFhd1 and EFhd2 in physiological processes.

## 1. Introduction

Actin is distributed in the cytosol and within some organelles and contributes to cell migration, division and trafficking as well as to the maintenance of the proper cell shape. All of these phenomena rely on F-actin, a filamentous polymer composed of G-actin monomers, which undergoes assembly, disassembly, severing, branching and bundling mediated by various actin-binding proteins (ABPs) during the course of its activity (Winder & Ayscough, 2005 ▸; Dos Remedios *et al.*, 2003 ▸). These ABPs all contain domains related to actin binding or regulation. The calponin homology (CH) domain is one of the most common modules. It consists of six α-helices (helix I–VI) and forms a compact structure through a network of hydro­phobic interactions (Yin *et al.*, 2020 ▸; Bramham *et al.*, 2002 ▸). The domain both mediates actin binding and serves a regulatory function (Gimona *et al.*, 2002 ▸). Another actin-related domain is the formin homology 2 (FH2) domain, an independently folding region conserved in the formin homology family (Higgs & Peterson, 2005 ▸; Wallar & Alberts, 2003 ▸). The mostly α-helical FH2 domain forms a unique dimer tethered together at either end (Xu *et al.*, 2004 ▸). It is able to influence actin dynamics and contribute to actin filament assembly and elongation (Higgs & Peterson, 2005 ▸).

Several ABPs also contain EF-hand motifs (EF-hands), which are helix–loop–helix structures, mostly found in pairs, within the hydro­phobic core of EF-hand domains (Day *et al.*, 2002 ▸; Denessiouk *et al.*, 2014 ▸). A number of EF-hand proteins undergo Ca^2+^-induced conformational changes that expose the hydro­phobic surface of each EF-hand domain, whereas others are much less affected by Ca^2+^. EF-hands are required for transducing Ca^2+^ signals into metabolic or mechanical responses and also serve to buffer Ca^2+^ levels within cells (Nelson & Chazin, 1998 ▸). Among the EF-hand protein superfamily, EFhd1 and EFhd2 are classified as ABPs. EFhd1 is localized within mitochondria, where it acts as a Ca^2+^-sensor for mitoflash activation (Tominaga *et al.*, 2006 ▸; Hou *et al.*, 2016 ▸). It is related to neuronal differentiation and pro/pre B-cell development (Tominaga *et al.*, 2006 ▸; Stein *et al.*, 2017 ▸). Neuronal energy homeostasis and mitochondrial morphology are influenced by both EFhd1 and β-actin (Xie *et al.*, 2018 ▸; Ulisse *et al.*, 2020 ▸). As an ABP within mitochondria, EFhd1 may be involved in regulating mitochondrial morphology via its Ca^2+^-dependent β-actin-bundling activity (Mun *et al.*, 2021 ▸).

EFhd2 is a cytoskeletal Ca^2+^-sensor protein localized in the cytosol (Purohit *et al.*, 2014 ▸). It was first detected in human CD8+ lymphocytes, where it stabilizes actin filaments by regulating the accessibility of F-actin to cofilin, which depol­ymerizes the F-actin (Vuadens *et al.*, 2004 ▸; Huh *et al.*, 2013 ▸). EFhd2 also regulates cytokine expression and lamellipodial dynamics through modulation of actin dynamics (Ramesh *et al.*, 2009 ▸; Kwon *et al.*, 2013 ▸), and the Ca^2+^-dependent actin-bundling activity of EFhd2 contributes to cell migration and spreading (Kwon *et al.*, 2013 ▸). In addition, EFhd2 may play an important role in several neurodegenerative diseases, as it is associated with tau in Alzheimer’s disease and other neurological disorders (Ferrer-Acosta *et al.*, 2013 ▸; Vega, 2016 ▸). Recently, it was found that EFhd2 is up-regulated in the cardiomyocytes during cardiac remodeling and repair (Giricz *et al.*, 2020 ▸). Although the functions and cellular localization of EFhd1 and EFhd2 differ, the two proteins exhibit a high degree of sequence identity (65%) (Mun *et al.*, 2021 ▸; Dutting *et al.*, 2011 ▸; Park *et al.*, 2016 ▸). The actin-bundling activity of EFhd2 requires Ca^2+^ for maintenance of the rigidity of EF-hands (Park *et al.*, 2016 ▸), and EFhd1 likely requires Ca^2+^ for actin bundling for the same reason.

Notably, Ca^2+^ is not the only metal that binds to EF-hands. For instance, Pb^2+^ reportedly activates calmodulin (CaM) and calcium binding protein (CaBP) by binding with higher affinity than Ca^2+^ (Fullmer *et al.*, 1985 ▸; Richardt *et al.*, 1986 ▸; Hui & Vogel, 1998 ▸). Moreover, the crystal structure of the Pb^2+^–CaM complex confirms that Pb^2+^ can substitute for Ca^2+^ within the EF-hands of CaM (Kursula & Majava, 2007 ▸). It is unknown, however, whether EF-hands in EFhd1 and/or EFhd2 bind Zn^2+^ or whether the Zn^2+^ binding affects their function, although we previously observed Zn^2+^-mediated multimerization of EFhd1 or EFhd2 via conserved residues at the crystal-packing interface of EFhd1. We previously reported EFhd1 and EFhd2 structures in the Ca^2+^-bound state (Mun *et al.*, 2021 ▸; Park *et al.*, 2016 ▸). Within the structure of EFhd1, however, we did not experimentally identify the metal ions, though both Ca^2+^ and Zn^2+^ were present under the crystallization conditions (Mun *et al.*, 2021 ▸).

Here, we report the structures of the mouse EFhd1 and human EFhd2 core domains in the Zn^2+^-bound state (EFhd1^Zn^, residues 79–180; EFhd2^Zn^, residues 82–180). We demonstrate Zn^2+^ coordination within the EF-hands of EFhd1 and EFhd2 through analysis of anomalous signals. Lastly, we show that Zn^2+^ affects the actin-bundling activities of EFhd1 and EFhd2 but not their actin binding, which suggests the possibility that Zn^2+^ acts to regulate EFhd1 and EFhd2 in both physiological and pathophysiological processes.

## 2. Results

### 2.1. Structures of Ca^2+^- or Zn^2+^-bound EFhd1 and EFhd2

**Figure 1:**
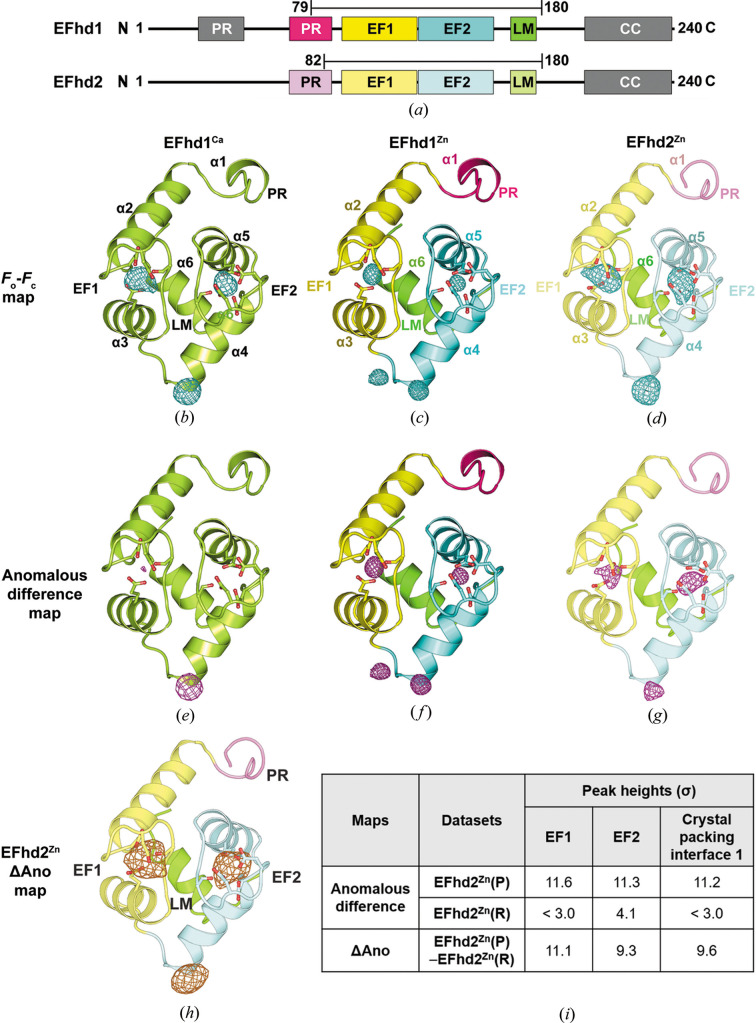
Analysis of σ_A_-weighted m*F*_o_ − D*F*_c_, anomalous difference and the difference between anomalous difference maps in EFhd1 or EFhd2. (*a*) Schematic of mouse EFhd1 and human EFhd2. Each is composed of a PR (proline-rich) region, EF-hand 1 (EF1), EF-hand 2 (EF2), LM (ligand mimic) helix and CC (coiled-coil) region. Upper bars indicate the crystallized regions of EFhd1^Ca^, EFhd1^Zn^ (residues, 79–180) and EFhd2^Zn^ (residues, 82–180). (*b*), (*c*), (*e*) and (*f*) Overall structure of EFhd1^Ca^ and EFhd1^Zn^. The EFhd1^Ca^ is shown in lime green. The EFhd1^Zn^ are shown in magenta, yellow, cyan or green. α1–6 indicates alpha helices 1–6. σ_A_-weighted *mF*_o_ − *DF*_c_ (*F*_o_ − *F*_c_) and anomalous difference maps are shown in cyan and magenta, respectively. (*d*) and (*g*) Overall structure of EFhd2^Zn^. The EFhd2^Zn^ are shown in magenta, yellow, cyan or green. The *F*_o_ − *F*_c_ and anomalous difference maps are shown in cyan and magenta, respectively. (*h*) Difference between anomalous difference maps (ΔAno) calculated from the EFhd2^Zn^(P) and EFhd2^Zn^(R) datasets. The ΔAno map was represented in the EFhd2 structure and shown in gold. (*i*) Table showing the peak heights (σ) of anomalous difference and ΔAno maps calculated from the EFhd2^Zn^(P) and EFhd2^Zn^(R) datasets. All maps are contoured at 3.0σ.

We previously reported the crystal structure of EFhd1 and suggested the occurrence of Zn^2+^-mediated multimerization of EFhd1 and EFhd2 (Mun *et al.*, 2021 ▸). On the other hand, the effects of Zn^2+^ on the actin-binding/bundling activities of EFhd1 and EFhd2 remain unknown. Therefore, to assess the role of Zn^2+^ in actin binding/bundling and its effect on the structures of the two proteins, we performed structural and biochemical studies with Zn^2+^-bound EFhd1 and EFhd2. To obtain EFhd1^Zn^, we added a final concentration of 1 m*M* CaCl_2_ to the protein solutions and used a crystallization buffer containing 2.5 m*M* ZnSO_4_. To obtain EFhd2^Zn^, we added EGTA and EDTA to final molar concentrations 15 times higher (0.18 m*M* each) than that of the protein to the EFhd2 proteins to remove native ions and then dialyzed the proteins. Thereafter, ZnCl_2_ was added to the protein solution to a final concentration of 0.75 m*M*. In the case of EFhd1^Ca^, CaCl_2_ was added to the protein solution directly to a final concentration of 4 m*M*. We crystallized these proteins and determined the crystal structures of EFhd1^Zn^, EFhd2^Zn^ and EFhd1^Ca^ at resolutions of 1.72, 2.60 and 2.80 Å, respectively, using molecular replacement methods [Table 1 ▸, Fig. 1 ▸(*a*)]. EFhd1^Zn^, EFhd2^Zn^ and EFhd1^Ca^ comprised two EF-hands, a ligand mimic (LM) helix at the C-terminus, a PR region at the N-terminus and a C-terminal linker (Mun *et al.*, 2021 ▸; Park *et al.*, 2016 ▸).

σ_A_-weighted m*F*_o_ − D*F*_c_ maps (*F*_o_ − *F*_c_ map) above 8σ were observed with these EFhd proteins, which suggests metal binding [Table 2 ▸, Figs. 1 ▸(*b*)–1(*d*)]. Considering our protein preparation protocols, we expected that the map originated from Ca^2+^ or Zn^2+^. To identify the coordinating metal, we calculated anomalous difference maps. Because Zn^2+^ and Ca^2+^ have different numbers of anomalous electrons at the wavelength the EFhd1 data were collected (Zn^2+^ *f*″ = 3.9 electrons, Ca^2+^ *f*″ = 0.9 electrons), the anomalous difference map should be weaker in the Ca^2+^-bound state than the Zn^2+^-bound state. In the case of EFhd1^Ca^, the peak heights in the anomalous difference map were 3.4 and 2.7σ for EF-hand 1 (EF1) and EF-hand 2 (EF2), respectively. EFhd1^Zn^ had peak heights of 23.0 and 14.0σ in the anomalous difference maps for EF1 and EF2, respectively. This suggests the metal coordinated in EFhd1^Ca^ was Ca^2+^ while that coordinated in EFhd1^Zn^ was Zn^2+^ [Table 3 ▸, Figs. 1 ▸(*c*) and 1 ▸(*f*)].

At a wavelength near the Zn *K*-edge (λ = 1.2851 Å/9648 eV), the Ca^2+^ *f*″ and Zn^2+^ *f*″ were 0.9 and 0.5 electrons, respectively. For that reason, we cannot rule out the possibility that the anomalous signals for EFhd2^Zn^ originated from Ca^2+^, because the wavelength for the EFhd2^Zn^ data collection was near the Zn *K*-edge [Fig. 1 ▸(*g*)]. To identify the metal coordinated in EFhd2, we analyzed the difference between anomalous difference maps (ΔAno). We used a single EFhd2^Zn^ crystal to collect datasets at the peak and remote positions of the Zn *K*-edge, which are termed EFhd2^Zn^(P) or EFhd2^Zn^(R), respectively [Table 4]. After calculation of the anomalous difference maps for each dataset, the anomalous difference map for EFhd2^Zn^(R) was subtracted from that for EFhd2^Zn^(P) to calculate the ΔAno map. The peak heights of the ΔAno map calculated from EFhd2^Zn^(P) and EFhd2^Zn^(R) were 11.1 and 9.3σ in EF1 and EF2, respectively [Figs. 1 ▸(*h*) and 1 ▸(*i*)]. This demonstrates that the metal coordinated in the EF-hands of EFhd2 was Zn^2+^.

Within the crystal structures of EFhd1^Ca^, EFhd1^Zn^ and EFhd2^Zn^, the anomalous difference maps were observed near the α4 of each protein, which is situated at the crystal-packing interface [Figs. 1 ▸(*e*)–1(*g*)]. Analyzing the metal coordination geometry using *CheckMyMetal* and the MetalPDB server, we expected that those peaks originated from Zn^2+^ (Zheng *et al.*, 2017 ▸; Putignano *et al.*, 2018 ▸). With EFhd1, the peak height in the anomalous difference map for the metal coordinated at the crystal-packing interface was 23.0σ in the case of EFhd1^Ca^. The metals coordinated at the crystal-packing interface of EFhd1^Zn^ had peak heights of 65.0 or 17.2σ in the anomalous difference map [Table 3 ▸, Figs. 1 ▸(*e*) and 1 ▸(*f*)]. In the case of EFhd2^Zn^, the peak height of the ΔAno map was 9.6σ at the crystal-packing interface [Figs. 1 ▸(*h*) and 1 ▸(*i*)]. Considering both the anomalous signals and the metal coordination geometry, we deemed the metal at the interface to be Zn^2+^. Collectively, the two EF-hands of EFhd1 and EFhd2 are able to coordinate Zn^2+^ as well as Ca^2+^, and Zn^2+^ could also be coordinated at the crystal-packing interface.

### 2.2. Comparison of the overall structures of Ca^2+^- and Zn^2+^-bound EFhd1 and EFhd2

**Figure 2:**
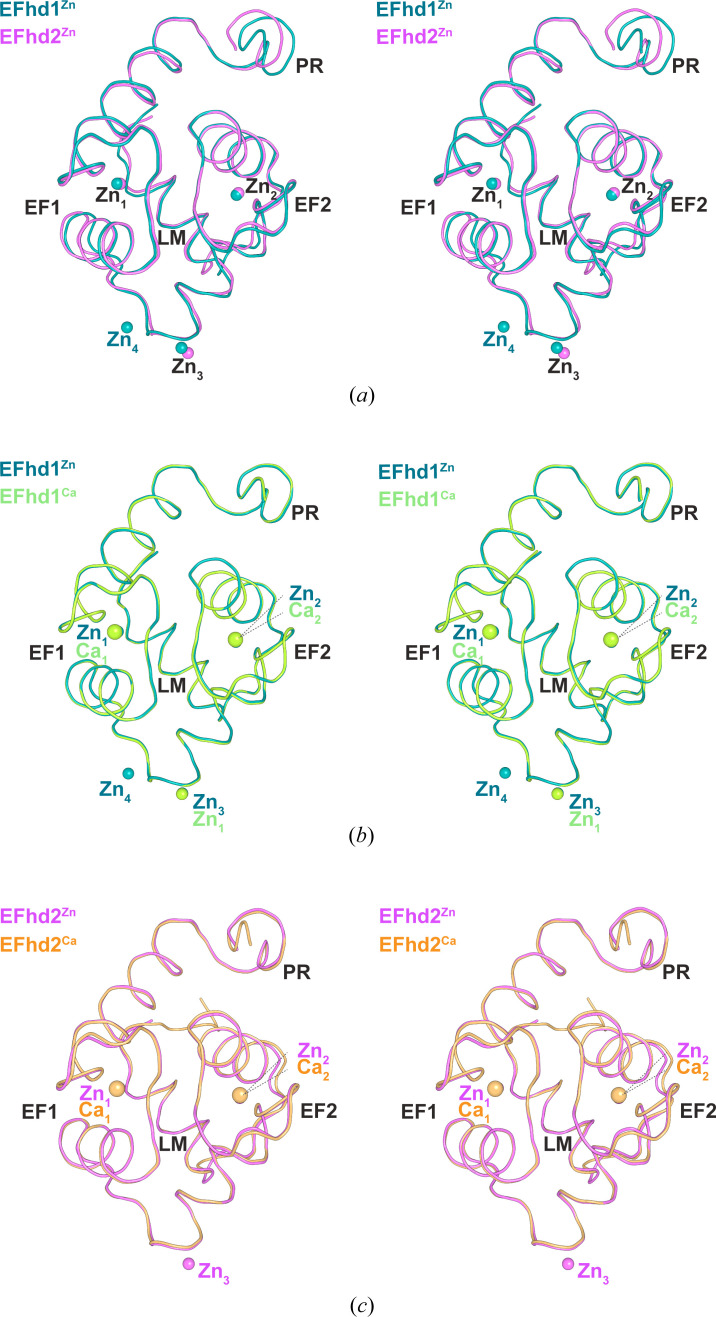
Superposition of overall structures of Ca^2+^- or Zn^2+^-bound EFhd1 and EFhd2. (*a*)–(*c*) Stereo diagrams of superimposed structures of EFhd1 and EFhd2 represented by ribbon diagrams. (*a*) Structural superposition of EFhd1^Zn^ in teal and EFhd2^Zn^ in magenta. (*b*) Structural superposition of EFhd1^Zn^ in teal and EFhd1^Ca^ in lime green. (*c*) Structural superposition of EFhd2^Zn^ in magenta and EFhd2^Ca^ in orange.

The overall structures of EFhd1^Zn^ and EFhd2^Zn^ superimposed well onto each other [the root mean square deviation (RMSD) of EFhd1^Zn^ and EFhd2^Zn^ was 0.381 Å for 80 C_α_ atoms; Fig. 2 ▸(*a*)]. Moreover, when we superimposed EFhd1^Zn^ on EFhd1^Ca^ and EFhd2^Zn^ on EFhd2^Ca^ (PDB entry 5i2l; Park *et al.*, 2016 ▸) to analyze the structural differences between the Zn^2+^- and Ca^2+^-bound states, we found that they too superimposed well [RMSD for EFhd1^Zn^ and EFhd1^Ca^ was 0.102 Å for 98 C_α_ atoms; RMSD for EFhd2^Zn^ and EFhd2^Ca^ was 0.224 Å for 87 C_α_ atoms; Figs. 2 ▸(*b*) and 2 ▸(*c*)] (Park *et al.*, 2016 ▸). Earlier findings suggest that EFhd1 and EFhd2 maintain open conformations irrespective of the presence of Ca^2+^ (Mun *et al.*, 2021 ▸; Park *et al.*, 2016 ▸). EFhd1^Zn^ and EFhd2^Zn^ also maintained open conformations similar to those of EFhd1^Ca^ and EFhd2^Ca^. Collectively, these results show that the overall structure of EFhd1^Zn^ is similar to that of EFhd2^Zn^, and the binding of Zn^2+^ has little effect on the conformations of the EFhd1 and EFhd2 core domains.

### 2.3. Structural comparison of the EF-hands in the Zn^2+^-bound EFhd1 and EFhd2 core domains

**Figure 3:**
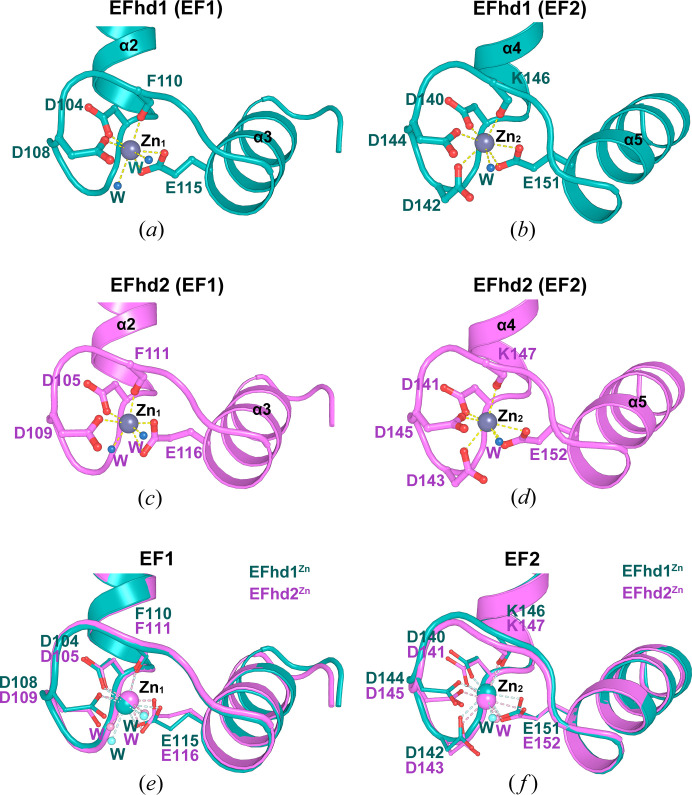
Structural comparisons of the EF-hands between EFhd1^Zn^ and EFhd2^Zn^. (*a*) and (*b*) Detailed illustrated views of (*a*) EF1 and (*b*) EF2 in EFhd1^Zn^ with Zn^2+^ bound. EF1 and EF2 are shown in teal. Zn^2+^ and water are shown as dark gray and sky blue spheres, respectively. (*c*) and (*d*) Detailed illustrated views of (*c*) EF1 and (*d*) EF2 in EFhd2^Zn^ with Zn^2+^ bound. EF1 and EF2 are shown in magenta. Zn^2+^ and water are shown as dark gray and sky blue spheres, respectively. (*e*) and (*f*) Detailed illustrated view of superimposed (*e*) EF1 and (*f*) EF2 from EFhd1^Zn^ and EFhd2^Zn^. Zn^2+^ is shown as teal (EFhd1) and magenta (EFhd2) spheres. Water molecules are showed as pale teal and pale magenta spheres. In all panels, the residues for Zn^2+^ coordination are shown in stick form, and Zn^2+^ coordination is represented by dashed lines.

Consensus residues for Ca^2+^ or Mg^2+^ coordination within EF-hands are at positions 1(X), 3(Y), 5(Z), 7(−Y), 9(−X) and 12(−Z). Conventionally, one water molecule participates in metal coordination at position 9(−X). These residues coordinate Ca^2+^ through seven ligands with pentagonal bipyramidal geometry and coordinate Mg^2+^ through six ligands with octahedral geometry (Lewit-Bentley & Réty, 2000 ▸; Grabarek, 2006 ▸; Nelson & Chazin, 1998 ▸). In EFhd1^Zn^ and EFhd2^Zn^, both EF-hands coordinated Zn^2+^. Zn_1_ and Zn_2_ in both EFhd1^Zn^ and EFhd2^Zn^ were each coordinated by seven oxygen atoms. In addition, two water molecules coordinated Zn_1_ at positions 3(Y) and 9(−X). The Gly at position 3(Y) (G106 in EFhd1, G107 in EFhd2) and the Asp at position 9(−X) (D112 in EFhd1, D113 in EFhd2) are not used to coordinate Zn_1_ [Figs. 3 ▸(*a*) and 3 ▸(*c*)]. Therefore, the geometry of the Zn_1_ coordination formed a distorted pentagonal bipyramid in the two proteins. Only one water coordinated Zn_2_ at position 9(−X) (S148 in EFhd1, S149 in EFhd2), which is consistent with the conventional one water coordination at that position [Figs. 3 ▸(*b*) and 3 ▸(*d*)]. As a result, the geometry of the Zn_2_ coordination formed the typical pentagonal bipyramid. To compare their EF-hands, we superimposed the structure EF1 or EF2 in EFhd1^Zn^ on EFhd2^Zn^ and found that both superimposed well [RMSD for EF1 in EFhd1^Zn^ and EFhd2^Zn^ was 0.300 Å for 32 C_α_ atoms; RMSD for EF2 in EFhd1^Zn^ and EFhd2^Zn^ was 0.456 Å for 36 C_α_ atoms; Figs. 3 ▸(*e*) and 3 ▸(*f*)]. In addition, measurement of the average distance between Zn^2+^ and its coordinating ligands in the EF-hands revealed that the overall average of Zn^2+^ coordination distances in EFhd1^Zn^ and EFhd2^Zn^ are similar to the average Zn^2+^–oxygen distance (2.3 ± 0.5 Å) (Table S1 of the supporting information) (Ireland & Martin, 2019 ▸).

### 2.4. Structural comparison of the EF-hands of the Zn^2+^- and Ca^2+^-bound states of EFhd1 or EFhd2

**Figure 4:**
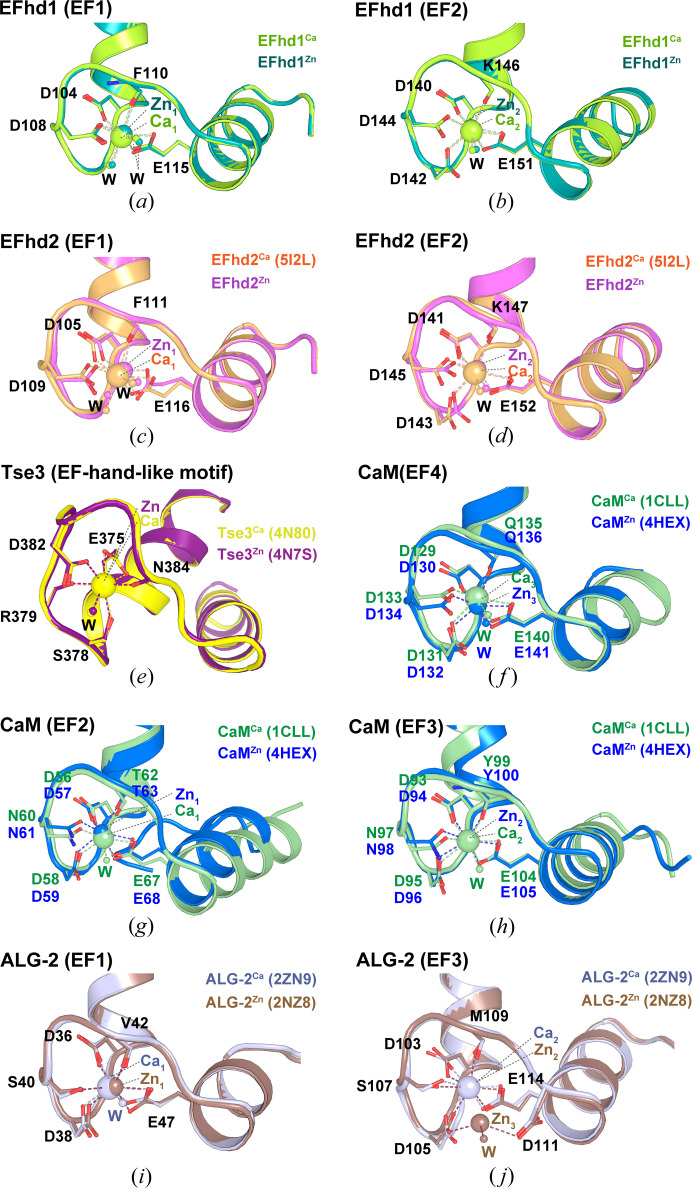
Structural comparison of Ca^2+^- and Zn^2+^-bound EF-hands among EFhd1, EFhd2 and other proteins. Detailed view of superimposed (*a*) EF-hand 1 (EF1) and (*b*) EF-hand 2 (EF2) in EFhd1^Ca^ and EFhd1^Zn^. EFhd1^Ca^ and EFhd1^Zn^ are shown in lime green and teal, respectively. Detailed view of superimposed (*c*) EF1 and (*d*) EF2 in EFhd2^Ca^ and EFhd2^Zn^. EFhd2^Ca^ (PDB entry 5i2l) and EFhd2^Zn^ are orange and magenta, respectively. Detailed view of the superimposed EF-hand-like motifs in (*e*) Tse3^Ca^ in yellow (PDB entry 4n80) and Tse3^Zn^ in purple (PDB entry 4n7s) and EF4 in (*f*) CaM^Ca^ in pale green (PDB entry 1cll) and CaM^Zn^ in blue (PDB entry 4hex). Detailed view of superimposed (*g*) EF2 and (*h*) EF-hand 3 (EF3) in CaM^Ca^ in pale green (PDB entry 1cll) and CaM^Zn^ in blue (PDB entry 4hex). Detailed view of superimposed (*i*) EF1 and (*j*) EF3 of ALG-2^Ca^ in silver (PDB entry 2zn9) and ALG-2^Zn^ in bronze (PDB entry 2zn8). In all panels, the residues for Zn^2+^ coordination are represented in stick form, and Zn^2+^ coordination is represented by dashed lines.

We next compared the structures of the EF-hands in the Ca^2+^- and Zn^2+^-bound states. When we superimposed the EF-hands of EFhd1^Ca^ and EFhd1^Zn^ and those of EFhd2^Ca^ and EFhd2^Zn^, they both superimposed well (RMSD for EF1 in EFhd1^Ca^ and EFhd1^Zn^ = 0.100 Å for 38 C_α_ atoms; RMSD for EF2 in EFhd1^Ca^ and EFhd1^Zn^ = 0.088 Å for 35 C_α_ atoms; RMSD for EF1 in EFhd2^Ca^ and EFhd2^Zn^ = 0.157 Å for 30 C_α_ atoms; and RMSD for EF2 in EFhd2^Ca^ and EFhd2^Zn^ = 0.253 Å for 36 C_α_ atoms). In addition, these proteins showed similar geometries in the Ca^2+^- and Zn^2+^-bound states. In both proteins EF1 and EF2 formed the distorted or typical pentagonal bipyramid for Ca^2+^ and Zn^2+^ coordination [Figs. 4 ▸(*a*)–4(*d*)]. The side-chain topologies of the EF-hand loop were similar between EFhd1^Zn^ and EFhd1^Ca^ and between EFhd2^Zn^ and EFhd2^Ca^. This suggests the metal coordination geometries of Ca^2+^ and Zn^2+^ are similar within EFhd proteins.

### 2.5. Structural comparison of the EF-hands in EFhd1^Zn^ and EFhd2^Zn^ with other Zn^2+^-bound proteins

EFhd proteins were able to coordinate Zn^2+^ as well as Ca^2+^ within their EF-hands. Similarly, several other proteins, including Tse3 and calmodulin (CaM), also coordinated Zn^2+^ within their EF-hand or EF-hand-like motif. In the case of Tse3, an EF-hand-like motif was able to coordinate Ca^2+^ or Zn^2+^, and the average coordination distance for Zn^2+^ is 2.5 Å, which is slightly longer than the average Zn^2+^–oxygen distance (2.3 ± 0.5 Å; Table S1) (Lu *et al.*, 2014 ▸). When we superimposed the EF-hand-like motifs of the Ca^2+^- and Zn^2+^-bound Tse3 structures [Tse3^Ca^ (PDB entry 4n80; Lu *et al.*, 2014 ▸), Tse3^Zn^ (PDB entry 4n7s; Lu *et al.*, 2014 ▸)], they were superimposed well, with an RMSD of 0.125 Å for 31 C_α_ atoms. Moreover, those EF-hand-like motifs had similar metal coordination geometries: a pentagonal bipyramid with seven ligands, including one water molecule [Fig. 4 ▸(*e*)]. This shows that the structure and metal coordination geometry of the EF-hand-like motif of Tse3 are comparable to those of EF2 of EFhd proteins but different from those of EF1.

CaM also coordinates both Zn^2+^ and Ca^2+^ within EF-hands [CaM^Zn^ (PDB entry 4hex; Kumar *et al.*, 2013 ▸), CaM^Ca^ (PDB entry 1cll; Chattopadhyaya *et al.*, 1992 ▸)]. Within the structure of CaM^Zn^, which comprises two chains (chains A and B) within the asymmetric unit, there are three Zn^2+^-bound EF-hands: EF4 in chain B and EF2 and EF3 in chain A. In the case of EF4, Ca^2+^ or Zn^2+^ is coordinated by seven ligands forming a general pentagonal bipyramid [Fig. 4 ▸(*f*)]. In EF2, seven oxygen atoms, including a water oxygen, coordinated Ca^2+^ (Ca_1_) with typical pentagonal bipyramidal geometry. Zn^2+^ (Zn_1_) was coordinated within EF2 of CaM^Zn^ by six oxygen atoms. Because there is no water in the Zn_1_ coordination, it formed a distorted pentagonal bipyramidal geometry with a vacancy at position 9(−X) [Fig. 4 ▸(*g*)]. In EF3 of CaM^Ca^, Ca_2_ coordination and geometry were the same as those in EF2 and formed the typical pentagonal bipyramid. Zn_2_ coordination and geometry in EF3 were also the same as those in EF2 and formed a distorted pentagonal bipyramid with the absence of a water at position 9(−X) [Fig. 4 ▸(*h*)]. The average metal coordination distances differed slightly between Ca^2+^ and Zn^2+^. The average coordination distances with Ca^2+^ in CaM EF4, EF2 and EF3 were 2.4, 2.3 and 2.4 Å, respectively; the average coordination distances with Zn^2+^ in these two EF-hands were 2.3 Å for EF4 and 2.4 Å for both EF2 and EF3 (Table S1). In CaM^Zn^, all of the average coordination distances for Zn^2+^ were around 2.3 ± 0.5 Å, the average Zn^2+^–oxygen distance. When we superimposed EF2 of CaM^Zn^ and CaM^Ca^ or EF3 of CaM^Zn^ and CaM^Ca^, the topologies of the alpha helices in CaM^Zn^ and CaM^Ca^ differed slightly in both cases [Figs. 4 ▸(*g*) and 4 ▸(*h*)]. Thus, there were no significant structural differences between the Ca^2+^- and Zn^2+^-bound EF-hands in EFhd proteins or EF4 of CaM. For EF2 and EF3 of CaM, however, differences between the Ca^2+^- and Zn^2+^-bound EF-hands were detected.

ALG-2 belongs to the penta-EF-hand (PEF) protein family and contains three metal binding EF-hands (Jia *et al.*, 2001 ▸). When we superimposed the EF-hands of Ca^2+^-bound and Zn^2+^-bound ALG-2 [ALG-2^Ca^ (PDB entry 2zn9; Suzuki *et al.*, 2008 ▸) and ALG-2^Zn^ (PDB entry 2zn8; Suzuki *et al.*, 2008 ▸)], they superimposed well [RMSD for EF1 = 0.265 Å for 31 C_α_ atoms; RMSD for EF3 = 0.292 Å for 35 C_α_ atoms; Figs. 4 ▸(*i*) and 4 ▸(*j*)]. Ca_1_ was coordinated by six oxygen atoms, including a water oxygen and excluding the S40 oxygen, and assumed a pentagonal bipyramidal geometry. In ALG-2^Zn^, Zn_1_ was coordinated by the six oxygen atoms in EF1 with the distorted pentagonal bipyramidal geometry [Fig. 4 ▸(*i*)]. Ca_2_ was also coordinated by six oxygen atoms in EF3 and formed a distorted pentagonal bipyramid. No water molecule was observed. On rare occasions, two Zn^2+^ ions, Zn_2_ and Zn_3_, simultaneously bound to EF3 in ALG-2^Zn^. Zn_2_ and Zn_3_ were coordinated by six and three oxygen atoms, forming a trigonal prism and distorted tetrahedron, respectively. Asp105 which participated in the Ca^2+^ coordination bound to Zn_2_ and Zn_3_ as a bidentate ligand, as did Glu114, and Asp111, which did not participate in Ca^2+^ coordination, bound to Zn_3_ [Fig. 4 ▸(*j*)]. The average coordination distances for Ca^2+^ in the two EF-hands were 2.4 and 2.5 Å, respectively; the average coordination distances for Zn^2+^ in EF1 and EF3 were the same as those for Ca^2+^, which are slightly longer than the average Zn^2+^–oxygen distance (Table S1). Therefore, the structures of the EF-hands in ALG-2^Zn^ were similar to those in ALG-2^Ca^, but the metal coordination of ALG-2^Zn^ showed diverse geometries unlike those of ALG-2^Ca^. These results show that the EF-hand structures and metal coordination of ALG-2^Ca^ are similar to those of EFhd1^Ca^ and EFhd2^Ca^, but those of ALG-2^Zn^ differ from those of EFhd1^Zn^ and EFhd2^Zn^.

Consequently, these suggested that the Ca^2+^ coordination geometry within EF-hands consistently formed a pentagonal bipyramid, whereas the Zn^2+^ coordination geometry assumed a variety of forms.

### 2.6. Zn^2+^-mediated actin-binding/bundling activities of EFhd1 and EFhd2

**Figure 5:**
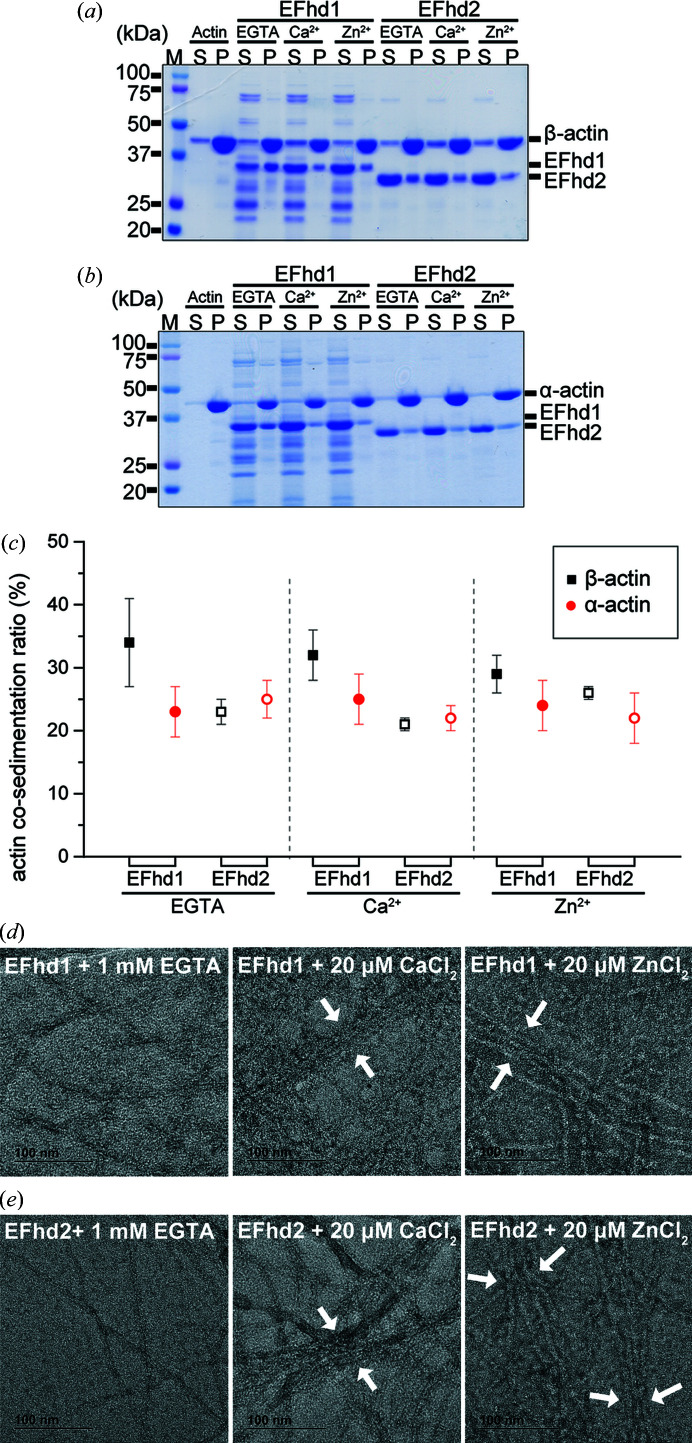
*In vitro* actin co-sedimentation assay (F-actin binding) and negative staining electron microscopy. SDS–PAGE analysis of *in vitro* actin co-sedimentation assays with EFhd1 and EFhd2. Protein samples (12 µ*M*) were added to polymerized (*a*) β-actin (8 µ*M*) or (*b*) α-actin (8 µ*M*) in the presence of 1 m*M* EGTA, 1 m*M* CaCl_2_ or 20 µM ZnCl_2_. (*c*) Co-sedimentation ratios from each experiment. Filled and open black squares show the co-sedimentation ratios for EFhd1 and EFhd2 with β-actin, respectively, while filled and open red spheres show the co-sedimentation ratios for EFhd1 and EFhd2 with α-actin, respectively. Symbols and error bars represent the mean and 95% confidence interval for the mean, which were calculated from five independent experiments. Negatively stained electron micrographs: (*d*) F-actin assembled from β-actin in the presence of EFhd1 and 1 m*M* EGTA, 20 µ*M* CaCl_2_ or ZnCl_2_; (*e*) F-actin assembled from α-actin in the presence of EFhd2 and 1 m*M* EGTA, 20 µ*M* CaCl_2_ or ZnCl_2_.

EFhd1 and EFhd2 both exhibit Ca^2+^-independent actin-binding and Ca^2+^-dependent actin-bundling activity (Mun *et al.*, 2021 ▸; Huh *et al.*, 2013 ▸; Kwon *et al.*, 2013 ▸). Because the EF-hands of EFhd1 and EFhd2 are situated within the actin-binding site, we hypothesized that Zn^2+^ may affect the actin-binding and/or bundling activities of EFhd1 and EFhd2 (Kwon *et al.*, 2013 ▸). To determine whether EFhd1 and/or EFhd2 bind F-actin in the presence of Zn^2+^, we performed *in vitro* high-speed co-sedimentation assays using α- and β-actin with full-length EFhd1 and EFhd2 [Figs. 5 ▸(*a*) and 5 ▸(*b*)]. As previously reported, EFhd1 and EFhd2 bound α- and β-actin independently of Ca^2+^ (Mun *et al.*, 2021 ▸). As reflected by the percentage bound, the binding affinity of β-actin for EFhd1 was higher than for EFhd2 (β-actin EGTA EFhd1: 34 ± 7%, β-actin EGTA EFhd2: 23 ± 2%, β-actin Ca^2+^ EFhd1: 32 ± 4% and β-actin Ca^2+^ EFhd2: 21 ± 1%), whereas the binding affinity of α-actin was similar for EFhd1 and EFhd2 (α-actin EGTA EFhd1: 23 ± 4%, α -actin EGTA EFhd2: 25 ± 3%, α-actin Ca^2+^ EFhd1: 25 ± 4% and α-actin Ca^2+^ EFhd2: 22 ± 2%) [Fig. 5 ▸(*c*)]. In the presence of Zn^2+^, EFhd1 and EFhd2 bound to F-actin and showed similar binding affinities for β- and α-actin (β-actin 20 µ*M* Zn^2+^ EFhd1: 29 ± 3%, β-actin 20 µ*M* Zn^2+^ EFhd2: 26 ± 1%, α-actin 20 µ*M* Zn^2+^ EFhd1: 24 ± 4%, and α-actin 20 µ*M* Zn^2+^ EFhd2: 22 ± 4%) [Fig. 5 ▸(*c*)]. Thus, both EFhd1 and EFhd2 bind actin in the presence of Ca^2+^, Zn^2+^ or EGTA. Consequently, actin binding is both Ca^2+^- and Zn^2+^-independent.

Lastly, we used electron microscopy with negative staining to assess whether Zn^2+^ affects the actin-bundling activities of EFhd1 and EFhd2. Because the subcellular localization of α-actin is in the cytosol and β-actin is in mitochondria, we separately analyzed the actin-bundling activities of EFhd1 and EFhd2 with β-actin and α-actin [Figs. 5 ▸(*d*) and 5 ▸(*e*)] (Xie *et al.*, 2018 ▸; Storch *et al.*, 2007 ▸; Reyes *et al.*, 2011 ▸). In the electron micrographs, we observed F-actin bundles in the presence of Ca^2+^ or Zn^2+^, but not in the presence of EGTA. We therefore conclude that EFhd1 and EFhd2 are capable of mediating Ca^2+^-dependent and Zn^2+^-dependent actin bundling.

## 3. Discussion

The Ca^2+^-binding proteins EFhd1 and EFhd2 regulate Ca^2+^-dependent F-actin bundling (Mun *et al.*, 2021 ▸; Kwon *et al.*, 2013 ▸; Park *et al.*, 2016 ▸). However, cells contain a variety of metals, and it is unknown whether metals other than Ca^2+^ also affect EFhd1 and EFhd2 function. In the present study, we determined the crystal structures of EFhd1 and EFhd2 coordinating Zn^2+^ within their EF-hands and at the crystal-packing interface. In addition, we determined that EFhd1 and EFhd2 bind actin independently of Ca^2+^ or Zn^2+^ and also exhibit Ca^2+^-dependent and Zn^2+^-dependent actin-bundling activity.

Smaller than Ca^2+^ (Zn: *r* = 0.74 Å versus Ca: *r* = 0.99 Å), Zn^2+^ contributes to diverse physiological functions (Kambe *et al.*, 2015 ▸; Allouche *et al.*, 1999 ▸). When Zn^2+^ interacts with proteins, Cys, His, Asp and Glu are frequently involved in its coordination (Vahrenkamp, 2007 ▸; Laitaoja *et al.*, 2013 ▸). Moreover, its lack of ligand field effects makes Zn^2+^ suitable for different coordination numbers and binding geometries in different biological settings (Laitaoja *et al.*, 2013 ▸). Through analysis of EF-hand structures, which are able to coordinate Ca^2+^ or Zn^2+^, it was found that the Zn^2+^ can be coordinated through more diverse metal coordination geometries than Ca^2+^ (Grabarek, 2006 ▸, Kumar *et al.*, 2013 ▸; Lu *et al.*, 2014 ▸; Suzuki *et al.*, 2008 ▸). Through anomalous signal analysis, we demonstrated that EFhd1 and EFhd2 are able to coordinate not only Ca^2+^ but also Zn^2+^ within their EF-hands. For the EFhd proteins, the binding of Ca^2+^ or Zn^2+^ had little effect on the conformations of EFhd1 or EFhd2, which probably explains why they are able to mediate actin bundling in the presence of Zn^2+^. In an earlier study, transmission electron microscopy revealed the presence of bundled actin filaments in ZnO-treated cells (Garcia-Hevia *et al.*, 2016 ▸). Because EFhd2 is localized in the cytosol, Zn^2+^-dependent actin bundling mediated by EFhd2 may have contributed to the bundled actin filaments detected in that study.

In resting cells, the cytosolic [Ca^2+^] is ∼100 n*M*, whereas the cytosolic [Zn^2+^] is tightly controlled in the picomolar to low nanomolar range (Kambe *et al.*, 2015 ▸; Esteras & Abramov, 2020 ▸; Patergnani *et al.*, 2020 ▸). In addition, the EF-hands of EFhd2 exhibit high Ca^2+^-binding affinities (*K*_d_ of EF1 = 96 ± 15 n*M*, *K*_d_ of EF2 = 70 ± 1 n*M*), implying that EFhd2 is likely to have Ca^2+^ bound within resting cells. The binding affinity of EFhd1 for Ca^2+^ has not been previously reported. We therefore measured the affinity of Ca^2+^ for EFhd1 using ITC (*K*_d_ = 22.3 ± 0.2 n*M*; Fig. S2 of the supporting information). The mitochondrial [Ca^2+^] is similar to that in the cytosol, whereas [Zn^2+^] is in the picomolar range under resting conditions (Kambe *et al.*, 2015 ▸; Esteras & Abramov, 2020 ▸; Patergnani *et al.*, 2020 ▸). Thus, EFhd1 may also mainly coordinate Ca^2+^ in resting cells due to the higher [Ca^2+^] than [Zn^2+^]. On the other hand, under conditions of Zn^2+^ overload, the mitochondrial [Zn^2+^] can reach the submicromolar range (Sensi *et al.*, 2003 ▸), and Zn^2+^-mediated multimerization of EFhd1 may occur, as we previously reported (Mun *et al.*, 2021 ▸).

EFhd2 is a novel amyloid protein that forms filaments using its coiled-coil region to self-oligomerize. EFhd2 and tau granules have been observed in fractions obtained from Alzheimer disease (AD) brains, suggesting a novel amyloid protein may form nucleation centers to induce the formation of tau aggregates (Ferrer-Acosta *et al.*, 2013 ▸). We previously suggested that Zn^2+^ mediates multimerization of EFhd1 and EFhd2 through protein aggregation. In addition, we confirmed that Zn^2+^ mediates crystal-packing interactions between EFhd2 molecules, which raises the possibility of the involvement of Zn^2+^-mediated multimerization in AD. Consistent with that idea, Zn^2+^ is reported to be significantly elevated in the AD neuropil (Lovell *et al.*, 1998 ▸). We therefore suggest that Zn^2+^ may be the seed for self-oligomerization of the novel amyloid protein EFhd2.

In the present study, we determined the crystal structures of EFhd1 and EFhd2 in the Zn^2+^-bound state, without the coiled-coil region. We also found that Zn^2+^-bound full-length EFhd1 and EFhd2 bind actin and mediate actin bundling. However, understanding the coiled-coil regions of EFhd1 and EFhd2 is important, given its association with self-oligomerization and actin-bundling activity (Kwon *et al.*, 2013 ▸; Ferrer-Acosta *et al.*, 2013 ▸). We therefore anticipate that structural studies of full-length EFhd1 and EFhd2 alone and in complex with actin will be useful for achieving a fuller understanding of the biological functions of these two proteins.

## 4. Materials and methods

### 4.1. Plasmid

Mouse EFhd1 ΔNTD (residues 69−240) and the human EFhd2 core domain (residues 70−184) were amplified from full-length mouse EFhd1 (residues 1−240) and human EFhd2 (residues 1−240), respectively, using polymerase chain reaction (PCR). The amplified EFhd1 ΔNTD was cloned into a modified pET28a vector (Novagen) containing an N-terminal His_6_ tag and a tobacco etch virus (TEV) protease cleavage site (Glu–Asn–Leu–Tyr–Phe–Gln/Gly). The amplified EFhd2 core domain was cloned into a modified pET41a vector containing gluta­thione S-transferase (GST) with a TEV protease cleavage site. Full-length EFhd1 was cloned into a modified pET28a vector (Novagen) with an N-terminal His_6_-TEV tag. Full-length EFhd2 was cloned into a modified pET28a vector carrying an N-terminal His_6_ tag.

### 4.2. Protein expression and purification of EFhd1 ΔNTD (residues 69−240)

Protein expression and purification of mouse EFhd1 ΔNTD were performed as reported previously (Mun *et al.*, 2021 ▸). The target protein was finally purified through a HiLoad 16/60 Superdex 75 gel-filtration column (GE Healthcare Life Sciences) pre-equilibrated with the final buffer [20 m*M* HEPES-NaOH (pH 7.5), 150 m*M* NaCl, 0.4 m*M* PMSF and 14.3 m*M* β-ME]. The purified protein was concentrated using a 10 K centrifugal filter (Millipore) and stored at −80°C. During purification, the presence of EFhd1 protein was confirmed using SDS–PAGE, and protein degradation was observed following incubation with TEV protease.

### 4.3. Protein expression and purification of EFhd2 core domain (residues 70−184)

Overall expression of the EFhd2 core domain was similar to that of EFhd1 ΔNTD. Cells transformed with the EFhd2 core domain were harvested by centrifugation, and the cell pellet was suspended in a lysis buffer [50 m*M* HEPES-NaOH (pH 7.5), 300 m*M* NaCl, 0.4 m*M* PMSF and 14.3 m*M* β-ME], lysed by sonication and centrifuged at 14 000*g* for 50 min at 4°C. The supernatant was then subjected to GST-bind agarose (Elpis) affinity chromatography. After washing with the lysis buffer, the target protein was eluted with lysis buffer supplemented with 30 m*M* gluta­thione, and the eluted protein was incubated with TEV protease overnight at 4°C to cleave the N-terminal GST-TEV tag. The target protein was further purified through a HiLoad 16/60 Superdex 75 gel-filtration column (GE Healthcare Life Sciences) pre-equilibrated with the final buffer [20 m*M* HEPES-NaOH (pH 7.5), 150 m*M* NaCl]. To obtain Zn^2+^-bound EFhd2 protein, the purified protein was treated with a 15-fold excess of EGTA and EDTA for 30 min at 4°C to remove pre-bound metal ions. The protein was then dialyzed in the final buffer for 24 h at 4°C, changing the buffer every 8 h. The dialyzed protein was concentrated using a 10 K centrifugal filter (Millipore) to 9.4 mg ml^−1^ and treated with 0.75 m*M* ZnCl_2_. The resultant Zn^2+^-bound protein was stored at −80°C.

### 4.4. Protein expression and purification of full-length EFhd1 and EFhd2

To investigate their actin-binding and bundling activities, we purified full-length EFhd1 and EFhd2. The protein expression and purification of EFhd1 and EFhd2 were performed as previously reported (Mun *et al.*, 2021 ▸). The two proteins were finally purified through a HiLoad 16/60 Superdex 75 gel-filtration column (GE Healthcare Life Sciences) pre-equilibrated with the final buffer containing 20 m*M* HEPES-NaOH (pH 7.5), 150 m*M* NaCl, 0.8 m*M* PMSF and 5 m*M* DTT. The purified protein was then concentrated using a 10 K centrifugal filter (Millipore) and stored at −80°C.

During purification, the presence of full-length EFhd1 and EFhd2 proteins was confirmed using SDS–PAGE.

### 4.5. Crystallization of the Ca^2+^- and Zn^2+^-bound EFhd1 core domain and Zn^2+^-bound EFhd2 core domain

We initially attempted to crystallize Ca^2+^- and Zn^2+^-bound EFhd1 ΔNTD (residues 69−240). Purified EFhd1 ΔNTD was incubated for at least 20 min on ice after the addition of 4 m*M* CaCl_2_ or 1 m*M* CaCl_2_ to 20.0 mg ml^−1^ and 11.2 mg ml^−1^ protein. Thereafter, 1 m*M* CaCl_2_ containing the protein was screened using the sitting-drop vapor-diffusion method in a 96-well sitting drop ‘IQ’ plate (SPT Labtech). We found that EFhd1 ΔNTD was degraded, and the core domain (residues 79−180) was crystallized. The EFhd1 core domain formed rod-shaped crystals after 1 week in reservoir solution containing 80 m*M* HEPES–NaOH (pH 7.0), 2 m*M* ZnSO_4_ and 25%(*v*/*v*) Jeffamine ED-2003 (Molecular Dimensions). Additional refinements of the crystallization conditions were performed using the sitting-drop vapor-diffusion method. Drops were prepared by mixing 1 µl of 1 m*M* CaCl_2_ containing the protein and 1 µl of reservoir solution or 3 µl of 4 m*M* CaCl_2_ containing the protein and 1 µl of reservoir solution. In the former mixing solution, the Zn^2+^-bound EFhd1 core domain (EFhd1^Zn^) crystals were obtained using reservoir solution containing 0.1 *M* Tris–HCl (pH 8.5), 2.5 m*M* ZnSO_4_ and 25%(*w*/*v*) Jeffamine ED-2001 (Hampton Research). In the latter mixing solution, crystals of Ca^2+^-bound EFhd1 core domain (EFhd1^Ca^) were obtained using reservoir solution containing 0.1 *M* Tris–HCl (pH 8.5), 0.4 m*M* ZnSO_4_ and 25% Jeffamine ED-2001 (Hampton Research). For data collection, crystals were cryoprotected by transferring them to mother liquor containing 30%(*v*/*v*) glycerol and flash freezing in liquid nitro­gen.

To obtain crystals of Zn^2+^-bound EFhd2 core domain (EFhd2^Zn^), we performed an initial screening using the sitting-drop vapor-diffusion method in a 96-well sitting drop ‘IQ’ plate (SPT Labtech). The EFhd2 core domain formed cubic crystals after 1 week in reservoir solution containing 0.1 *M* HEPES-NaOH (pH 7.5), 25%(*w*/*v*) PEG 8000. Additional refinements of the crystallization conditions were performed using the sitting-drop vapor-diffusion method with drops prepared by mixing 1 µl of protein and 1 µl of reservoir solution. Zn^2+^-bound EFhd2 core domain crystals were obtained using the same reservoir solution used for the initial screening. For data collection, crystals were cryoprotected by transferring them to mother liquor containing 20% glycerol and 1 m*M* ZnCl_2_ and flash freezing in liquid nitro­gen.

### 4.6. X-ray data collection, structure determination and refinement

X-ray diffraction data for EFhd1^Ca^, EFhd1^Zn^ and EFhd2^Zn^ were collected at 100 K using synchrotron X-ray sources on beamline 5C at the Pohang Accelerator Laboratory (PAL, South Korea). Ultimately, we collected the best resolution diffraction data for EFhd1^Ca^, EFhd1^Zn^ and EFhd2^Zn^ at 2.80, 1.72 and 2.60 Å resolution, respectively. Diffraction data were collected at wavelengths corresponding to the peak position of the Zn *K*-edge (λ = 1.2826 Å/9669 eV) or near the Zn *K*-edge (λ = 1.2851 Å/9648 eV). The crystals belonged to the space group *P*2_1_2_1_2_1_ (*a* = 44.3, *b* = 47.9 and *c* = 63.4 Å for EFhd1^Ca^; *a* = 44.2, *b* = 47.5 and *c* = 63.7 Å for EFhd1^Zn^; and *a* = *b* = *c* = 92.8 Å for EFhd2^Zn^; with α = β = γ = 90°). The diffraction data for EFhd1^Ca^ and EFhd1^Zn^ were indexed, processed and scaled using the *HKL2000* suite (Otwinowski & Minor, 1997 ▸). The diffraction data for EFhd2^Zn^ were indexed and integrated using *DIALS*/*xia2* in *CCP4i2*, and data merging and scaling were performed using *AIMLESS* from *CCP4* (Winter *et al.*, 2018 ▸; Evans & Murshudov, 2013 ▸; Winn *et al.*, 2011 ▸; Winter, 2010 ▸; Potterton *et al.*, 2018 ▸). Molecular replacement was carried out using *Phaser-MR* in the *Phenix* program suite, using the structures of the EFhd1 (PDB entry 7clt; Mun *et al.*, 2021 ▸) and EFhd2 core domains (PDB entry 5i2l) as the templates (Mun *et al.*, 2021 ▸; Park *et al.*, 2016 ▸; McCoy *et al.*, 2007 ▸; Liebschner *et al.*, 2019 ▸). Additional model building was performed using the program *Coot* (Emsley & Cowtan, 2004 ▸). Iterative refinement was performed with *phenix.refine* (Liebschner *et al.*, 2019 ▸; Afonine *et al.*, 2012 ▸). The N- and C-terminals of EFhd1^Ca^, EFhd1^Zn^ and EFhd2^Zn^ were partially disordered. Details of the data collection and refinement statistics are provided in Table 1 ▸.

### 4.7. Anomalous X-ray diffraction data collection at peak and remote positions of the Zn *K*-edge

To confirm Zn^2+^-binding by the EF-hands of EFhd2^Zn^, we collected diffraction data at wavelengths corresponding to the peak (λ = 1.2823 Å/9669 eV) and low-energy remote positions of the Zn *K*-edge (λ = 1.2917 Å/9599 eV). The diffraction data for EFhd2^Zn^ [data title: EFhd2^Zn^(P), EFhd2^Zn^(R)] were indexed, processed and scaled using the *HKL2000* suite (Otwinowski & Minor, 1997 ▸). Molecular replacement was carried out with *Phaser-MR* in the *Phenix* program suite, using the structures of the EFhd2 core domain (PDB entry 5i2l) as the template (McCoy *et al.*, 2007 ▸; Liebschner *et al.*, 2019 ▸). We created difference between anomalous difference maps (ΔAno) using the program *Coot* (Emsley & Cowtan, 2004 ▸). Details of the data collection and refinement statistics are provided in Table 4 ▸.

### 4.8. Structural analysis

All structural figures were generated using *PyMOL* (version 1.8.6.0; Schrödinger LLC). The σ_A_-weighted *mF*_o_ − *DF*_c_, anomalous difference and ΔAno maps were converted to the *CCP4* format using *phenix.maps* tools (Liebschner *et al.*, 2019 ▸; Pražnikar *et al.*, 2009 ▸) and were visualized in *PyMOL*.

### 4.9. Measurement of the Ca^2+^ binding affinity of wild-type EFhd1 using ITC

To assess the Ca^2+^ binding affinity of the EFhd1 core domain, we purified EFhd1 (69–200), which is more stable than EFhd1 ΔNTD or the full-length protein. For protein expression and purification of EFhd1 (69–200), the affinity chromatography, His_6_–TEV tag cleavage and gel-filtration steps were same as those used for EFhd1 ΔNTD, except the final buffer contained 20 m*M* HEPES–NaOH (pH 7.5) and 150 m*M* NaCl. After purification, EFhd1 (69−200) was treated with a tenfold excess of EGTA and EDTA for 30 min at 4°C to remove pre-bound metal ions. The protein was then dialyzed for 24 h at 4°C in buffer containing 50 m*M* Tris–HCl (pH 8.5) and 20 m*M* NaCl. The dialyzed EFhd1 (69–200) was again treated for 30 min at 4°C with a tenfold excess of EGTA and EDTA, after which the protein was again dialyzed for 24 h using the same dialysis buffer, which was refreshed every 8 h. The dialyzed protein was concentrated to 20 µ*M*, and the ligand solution (0.3 m*M* CaCl_2_) was prepared in the same buffer. Each EFhd1 (69–200) sample was titrated with 30 injections of ligand (6 µl) in a VP-ITC calorimeter (MicroCal). All measurements were carried out at 20°C, and binding isotherm analysis and fitting were conducted using the *Origin* software supplied with the calorimeter.

### 4.10. *In vitro* actin-binding assay

Actin co-sedimentation assays were performed as previously described (Mun *et al.*, 2021 ▸; Kwon *et al.*, 2013 ▸). In brief, non-muscle actin (85% β-actin and 15% γ-actin) derived from human platelets and muscle actin (α-actin) derived from rabbit skeletal muscle (Cytoskeleton Inc.) were mixed in G-buffer [0.2 m*M* CaCl_2_, 5 m*M* Tris–HCl (pH 8.0)] to produce actin stock and were polymerized in an actin polymerization buffer [100 m*M* KCl, 2 m*M* MgCl_2_, 0.5 m*M* ATP, 0.2 m*M* Tris–HCl (pH 8.0)] for 1 h at 24°C. Solutions (50 µl) containing polymerized actin (8 µ*M*) were incubated with EFhd1 (12 µ*M*) or EFhd2 (12 µ*M*) for 30 min at 24°C in the presence of 1 m*M* EGTA, 1 m*M* CaCl_2,_ or 20 µ*M* ZnCl_2_. Actin filaments with each protein were pelleted by centrifugation at 100 000*g* for 2 h at 24°C (for the actin-binding assay). Equal amounts of pellet and supernatant were resolved with SDS–PAGE, and the protein bands were visualized by Coomassie Blue staining. The percentage of each protein in the pellet was quantified with densitometry using *ImageJ* version 1.53*k*, and a percentage of pellet histogram was plotted using the *OriginPro* software (version 9.1; OriginLab Corporation, Northampton, MA, USA; Schneider *et al.*, 2012 ▸).

### 4.11. Negative-staining electron microscopy imaging

Muscle and non-muscle actin (Cytoskeleton Inc.) were polymerized in F-actin buffer containing 100 m*M* KCl, 2 m*M* MgCl_2_, 0.5 m*M* ATP and 0.2 m*M* Tris–HCl at pH 8.0. Mixtures (50 µl) of F-actin (4 µ*M*) and full-length EFhd1 (6 µ*M*) or EFhd2 (6 µ*M*) in the presence of 1 m*M* EGTA, 20 µ*M* CaCl_2_ or 20 µ*M* ZnCl_2_ were allowed to react for 1 h. For grid preparation, 2 µl of reaction mixture were loaded onto C-flat holey gold grids (CF-1.2/1.3-4Au-50) and blotted with filter paper to remove excess sample. The sample-loaded grid was then stained in a solution of 1%(*w*/*v*) uranyl acetate. The grids were immersed in the stain solution for 20 min, blotted with filter paper to remove excess stain and air-dried. The samples were imaged using an FEI Tecnai G2 F30 S-Twin transmission electron microscope operated at 300 kV.

## Supplementary Material

Supporting figures and table. DOI: 10.1107/S2052252523001501/jt5066sup1.pdf

PDB reference: Ca^2+^-bound EFhd1/Swiprosin-2, 7ygv

PDB reference: Zn^2+^-bound EFhd2/Swiprosin-1, 7ygy

PDB reference: Zn^2+^-bound EFhd1/Swiprosin-2, 7ygw

## Figures and Tables

**Table 1:** Data collection and refinement statistics Data collection was carried out at Beamline 5C at Pohang Accelerator Laboratory (PAL), Republic of Korea.

Data collection			
Dataset	EFhd1^Ca^	EFhd1^Zn^	EFhd2^Zn^
Final Ca^2+^ conc. (m*M*)	3	0.5	–
Final Zn^2+^ conc. (m*M*)	0.1	1.3	0.4
Space group	*P*2_1_2_1_2_1_	*P*2_1_2_1_2_1_	*I*23
X-ray source	PAL-5C	PAL-5C	PAL-5C
Detector	EIGER 9M	EIGER 9M	EIGER 9M
Wavelength (Å)	1.2826	1.2826	1.2851
Unit cell parameters			
*a*, *b*, *c* (Å)	44.3, 47.9, 63.4	44.0, 47.4, 63.4	92.8, 92.8, 92.8
α, β, γ (°)	90.0, 90.0, 90.0	90.0, 90.0, 90.0	90.0, 90.0, 90.0
Resolution range (Å)†	50–2.80 (2.85–2.80)	50–1.72 (1.75–1.72)	50–2.60 (2.72–2.60)
*R*_merge_ ‡	16.4 (49.2)	7.9 (82.9)	9.6 (133.9)
CC_1/2_	0.999 (0.959)	0.998 (0.774)	1.00 (0.960)
〈*I*/σ(*I*)〉	20.0 (3.7)	24.4 (2.1)	40.3 (4.0)
Completeness (%)	99.8 (98.3)	99.5 (94.5)	100.0 (100.0)
Redundancy§	22.0 (10.0)	12.5 (9.8)	80.8 (82.0)

Refinement			
Resolution range (Å)	31.7–2.80	38.0–1.72	46.5–2.60
No. of unique reflections	3614	14742	4243
*R*_work_/*R*_free_ (%)¶	19.5/25.2	19.8/21.1	22.9/26.0
*B* factors (Å^2^) of protein	38.9	22.3	67.2
No. atoms (residues)			
Protein	827 (102)	813 (102)	775 (99)
Glycerol	6 (1)	6 (1)	0
Ca^2+^	2	0	0
Zn^2+^	1	4	3
Water	20	32	5
Model statistics			
RMSD bond length (Å)	0.009	0.009	0.010
RMSD bond angle (°)	1.039	0.984	1.184
Ramachandran plot (%)			
Favored	99.0	99.0	95.9
Allowed	1.0	1.0	4.1
Disallowed	0.0	0.0	0.0
PDB entry	7ygv	7ygw	7ygy

† Values in parentheses are for the highest resolution shell.

‡ *R*_merge_ = ∑*_h_* ∑*_i_* |*I*(*h*)*_i_* − 〈*I*(*h*)〉|/[∑*_h_* ∑*_i_ I*(*h*)*_i_*], where *I*(*h*) is the intensity of the reflection of *h*, ∑*_h_* is the sum over all reflections and ∑*_i_* is the sum over *i* measurements of reflection *h*.

§ Redundancy: we collected EFhd1^Ca^ and EFhd2^Zn^ datasets using 720 frames, and EFhd1^Zn^ datasets using 360 frames due to the radiation decay.

¶ *R*_work_ = ∑*_hkl_* ||*F*_o_|−|*F*_c_||/(∑*_hkl_* |*F*_o_|); 5% of the reflections were excluded for the *R*_free_ calculation.

**Table 2:** Peak heights of the σ_A_-weighted *mF*_o_ − *DF*_c_ (*F*_o_ − *F*_c_) map of EFhd1^Ca^, EFhd1^Zn^ and EFhd2^Zn^

Dataset	Final metal concentration†	Data collection wavelength (Å)	Coordinated metal and peak heights of *F*_o_ − *F*_c_ map (σ)					
			EF-hand 1 (EF1)		EF-hand 2 (EF2)		Crystal-packing interface	
EFhd1^Ca^	3 m*M* CaCl_2_, 0.1 m*M* ZnSO_4_	1.2826‡	Ca_1_	12.6	Ca_2_	12.3	Zn_1_	17.0
EFhd1^Zn^	0.5 m*M* CaCl_2_, 1.3 m*M* ZnSO_4_	1.2826 ‡	Zn_1_	22.4	Zn_2_	24.6	Zn_3_	30.4
							Zn_4_	8.1
EFhd2^Zn^	0.4 m*M* ZnCl_2_	1.2851 §	Zn_1_	11.5	Zn_2_	10.3	Zn_3_	14.9

† Final concentration of CaCl_2_, ZnSO_4_ or ZnCl_2_ under crystallization conditions.

‡ Zn *K*-edge.

§ Near Zn *K*-edge.

**Table 3:** Peak heights of the anomalous difference map of EFhd1^Ca^, EFhd1^Zn^ and EFhd2^Zn^

Dataset	Final metal concentration†	Data collection wavelength (Å)	Coordinated metal and peak heights of anomalous difference map (σ)					
			EF-hand 1 (EF1)		EF-hand 2 (EF2)		Crystal-packing interface	
EFhd1^Ca^	3 m*M* CaCl_2_, 0.1 m*M* ZnSO_4_	1.2826‡	Ca_1_	3.4	Ca_2_	2.7	Zn_1_	23.0
EFhd1^Zn^	0.5 m*M* CaCl_2_, 1.3 m*M* ZnSO_4_	1.2826‡	Zn_1_	23.0	Zn_2_	14.0	Zn_3_	65.0
							Zn_4_	17.2
EFhd2^Zn^	0.4 m*M* ZnCl_2_	1.2851[Table-fn tfn10]	Zn_1_	5.3	Zn_2_	7.5	Zn_3_	7.0

† Final concentration of CaCl_2_, ZnSO_4_ or ZnCl_2_ under crystallization conditions.

‡ Zn *K*-edge.

§ Near Zn *K*-edge.

**Table 4:** The datasets for analyzing the anomalous signals at the peak or remote positions of the Zn *K*-edge The datasets were collected at peak [EFhd2^Zn^(P)] or remote [EFhd2^Zn^(R)] positions of the Zn *K*-edge using a single EFhd2^Zn^ crystal, and were used to analyze the anomalous signals described in Fig. 1 ▸.

Dataset	EFhd2^Zn^(P)	EFhd2^Zn^(R)
Space group	*I*23	*I*23
X-ray source	PAL-7A	PAL-7A
Detector	ADSC Q270	ADSC Q270
Wavelength (Å)	1.2823 (peak)	1.2917 (remote)
Unit cell parameters		
*a*, *b*, *c* (Å)	92.5, 92.5, 92.5	92.8, 92.8, 92.8
α, β, γ (°)	90.0, 90.0, 90.0	90.0, 90.0, 90.0
Resolution range (Å)	50–3.45 (3.51–3.45)	50–3.94 (4.01–3.94)
*R*_merge_	26.0 (192.8)	23.2 (184.7)
CC_1/2_	0.979 (0.898)	0.990 (0.939)
〈*I*/σ(*I*)〉	49.0 (5.0)	52.8 (6.9)
Completeness (%)	99.9 (100.0)	100.0 (100.0)
Redundancy	41.7 (43.5)	40.9 (42.8)
