# Supplementary material for: Structural and biochemical insights into Zn2+-bound EF-hand proteins, EFhd1 and EFhd2
Source: IUCrJ. 2023 Mar 1;10(Pt 2):233–45. doi: 10.1107/S2052252523001501 (PMC9980392; doi:10.1107/S2052252523001501)
Supplement: Supplementary file 1 [file m-10-00233-sup1.pdf]

IUCrJ

Volume 10 (2023)

Supporting information for article:

Structural and biochemical insights into Zn^{2+}-bound EF-hand proteins, EFhd1 and EFhd2

Sang A Mun, Jongseo Park, Jung Youn Kang, Taein Park, Minwoo Jin, Jihyeong Yang and Soo Hyun Eom

Table S1.

Distances between ligands within EF-hand motifs and metals (Ca^{2+} or Zn^{2+})

	**EFhd1**					
	**EF1**			**EF2**		
	AA^{atom}	Distance (Å)		AA^{atom}	Distance (Å)	
		Ca_1	Zn_1		Ca_2	Zn_2
**1 (X)**	$D104^{OD1}$	2.2	2.2	$D140^{OD1}$	2.3	2.1
**3 (Y)**	H_2O	2.3	2.4	$D142^{OD1}$	2.3	2.3
**5 (Z)**	$D108^{OD1}$	2.5	2.3	$D144^{OD1}$	2.4	2.3
**7 (-Y)**	$F110^{O}$	2.5	2.2	$K146^{O}$	2.5	2.3
**9 (-X)**	H_2O	2.3	2.4	H_2O	2.2	2.4
**12 (-Z)**	$E115^{OE1}$	2.4	2.4	$E151^{OE1}$	2.5	2.4
	$E115^{OE2}$	2.5	2.6	$E151^{OE2}$	2.7	2.7
	average	2.4	2.4	average	2.4	2.4

	**EFhd2**					
	**EF1**			**EF2**		
	AA^{atom}	Distance (Å)		AA^{atom}	Distance (Å)	
		Ca_1	Zn_1		Ca_2	Zn_2
**1 (X)**	$D105^{OD1}$	2.6	2.2	$D141^{OD1}$	2.6	2.3
**3 (Y)**	H_2O	2.5	2.0	$D143^{OD1}$	2.6	2.4
**5 (Z)**	$D109^{OD1}$	2.5	2.4	$D145^{OD1}$	2.6	2.1
**7 (-Y)**	$F111^{O}$	2.5	2.1	$K147^{O}$	2.6	2.5
**9 (-X)**	H_2O	2.5	2.0	H_2O	2.6	2.1
**12 (-Z)**	$E116^{OE1}$	2.6	2.0	$E152^{OE1}$	2.6	2.5
	$E116^{OE2}$	2.7	2.8	$E152^{OE2}$	2.7	2.5
	average	2.6	2.2	average	2.6	2.3

**Tse3**		
**EF-hand-like motif**		
**AA^{atom}**	Distance (Å)	
	Ca_1	Zn_1
**E375^{OE1}**	2.6	2.6
**E375^{OE2}**	2.6	2.7
**S378^{O}**	2.7	2.6
**R379^{O}**	2.4	2.3
**D382^{OD1}**	2.5	2.6
**N384^{O}**	2.5	2.4
**H_2O**	2.5	2.6
**average**	2.5	2.5

	**CaM**					
	**EF2**			**EF3**		
	AA^{atom}	Distance (Å)		AA^{atom}	Distance (Å)	
		Ca_1	Zn_1		Ca_2	Zn_2
**1 (X)**	D56^{OD1} (D57^{OD1})	2.1	2.0	D93^{OD1} (D94^{OD1})	2.2	2.2
**3 (Y)**	D58^{OD1} (D59^{OD1})	2.3	2.5	D95^{OD1} (D96^{OD1})	2.5	2.6
**5 (Z)**	N60^{OD1} (N61^{OD1})	2.4	2.3	N97^{OD1} (N98^{OD1})	2.3	2.6
**7 (-Y)**	T62^{O} (T63^{O})	2.3	2.4	Y99^{O} (Y100^{O})	2.2	2.3
**9 (-X)**	H_2O	2.3	-	H_2O	2.6	-
**12 (-Z)**	E67^{OE1} (E68^{OE1})	2.6	2.6	E104^{OE1} (E105^{OE1})	2.4	2.4
	E67^{OE2} (E68^{OE2})	2.4	2.6	E104^{OE2} (E105^{OE2})	2.6	2.5
	average	2.3	2.4	average	2.4	2.4

	**CaM**		
	**EF4**		
	AA^{atom}	Distance (Å)	
		Ca_3	Zn_3
**1 (X)**	$D129^{OD1}$ ($D130^{OD1}$)	2.3	2.2
**3 (Y)**	$D131^{OD1}$ ($D132^{OD1}$)	2.4	2.6
**5 (Z)**	$D133^{OD1}$ ($D134^{OD1}$)	2.3	2.3
**7 (-Y)**	$Q135^{O}$ ($Q136^{O}$)	2.3	2.3
**9 (-X)**	H_2O	2.6	2.1
**12 (-Z)**	$E140^{OE1}$ ($E141^{OE1}$)	2.6	2.4
	$E140^{OE2}$ ($E141^{OE2}$)	2.6	2.5
	average	2.4	2.3

	**ALG-2**					
	**EF1**			**EF3**		
	AA^{atom}	Distance (Å)		AA^{atom}	Distance (Å)	
		Ca_1	Zn_1		Ca_2	Zn_2
**1 (X)**	$D36^{OD1}$	2.1	2.2	$D103^{OD1}$	2.3	2.3
**3 (Y)**	$D38^{OD1}$	2.3	2.3	$D105^{OD1}$	2.6	2.4
**5 (Z)**	$S40^{OD1}$	-	2.4	$S107^{OD1}$	2.7	2.8
**7 (-Y)**	$V42^{O}$	2.5	2.1	$M109^{O}$	2.3	2.5
**9 (-X)**	H_2O	2.3	-	-	-	-
**12 (-Z)**	$E47^{OE1}$	2.9	2.5	$E114^{OE1}$	2.4	2.4
	$E47^{OE2}$	2.5	2.8	$E114^{OE2}$	2.4	2.8
	average	2.4	2.4	average	2.5	2.5

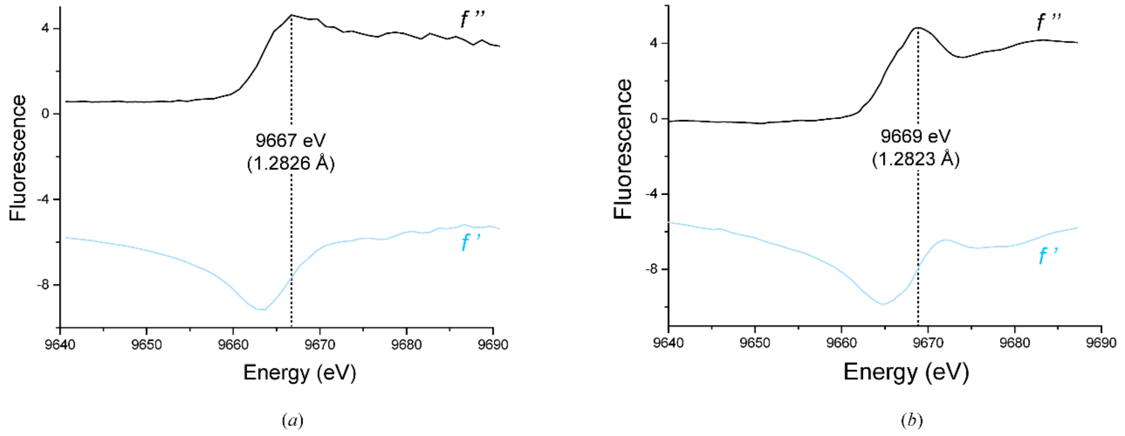

Figure S1. X-ray absorption scan of EFhd1^{Zn} and EFhd2^{Zn}(P) at the Zn K-edge region

(a,b) The plots show X-ray absorption scan of EFhd1^{Zn} or EFhd2^{Zn}(P) at the Zn K-edge. The energy and wavelengths of the peak positions are indicated on the dashed line in each plot.

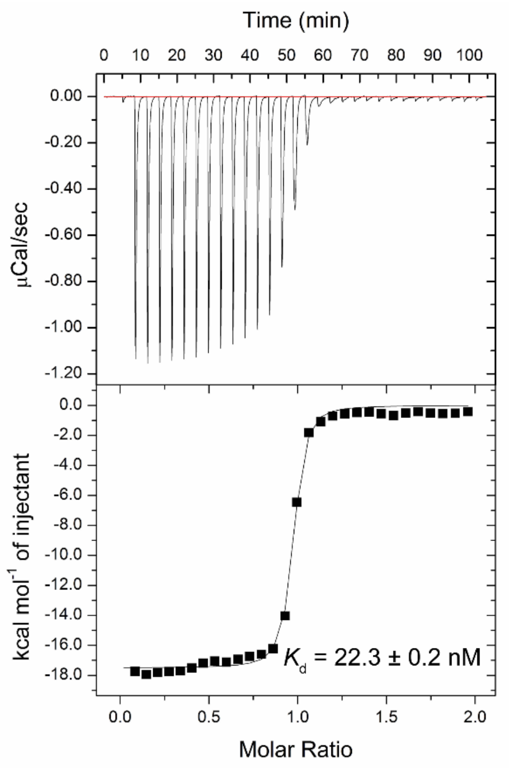

Figure S2. Measurement of the Ca^{2+} binding affinity for wild-type EFhd1 determined with ITC

ITC profile for wild-type EFhd1 with Ca^{2+}: 20 µM EFhd1 (residues 69–200) was titrated with 30 injections of 0.3 mM $CaCl_2$ at 20°C. The experiment was repeated in triplicate.
